# Effects of *Echium plantagineum* L. Bee Pollen on Basophil Degranulation: Relationship with Metabolic Profile

**DOI:** 10.3390/molecules190710635

**Published:** 2014-07-22

**Authors:** Eduarda Moita, Carla Sousa, Paula B. Andrade, Fátima Fernandes, Brígida R. Pinho, Luís R. Silva, Patrícia Valentão

**Affiliations:** REQUIMTE/Laboratório de Farmacognosia, Departamento de Química, Faculdade de Farmácia, Universidade do Porto, Rua de Jorge Viterbo Ferreira nº 228, 4050-313 Porto, Portugal; E-Mails: emoita07@hotmail.com (E.M.); csousa@ff.up.pt (C.S.); pandrade@ff.up.pt (P.B.A.); mfgfernandes@gmail.com (F.F.); brigidarpinho@gmail.com (B.R.P.); lmsilva@ff.up.pt (L.R.S.)

**Keywords:** *Echium plantagineum* L. bee pollen, RBL-2H3 cells degranulation, β-hexosaminidase, lipoxygenase, organic acids, fatty acids

## Abstract

This study aimed to evaluate the anti-allergic potential of *Echium plantagineum* L. bee pollen and to characterize its primary metabolites. The activity of *E. plantagineum* hydromethanolic extract, devoid of alkaloids, was tested against β-hexosaminidase release in rat basophilic leukemic cells (RBL-2H3). Two different stimuli were used: calcium ionophore A23187 and IgE/antigen. Lipoxygenase inhibitory activity was evaluated in a cell-free system using soybean lipoxygenase. Additionally, the extract was analysed by HPLC-UV for organic acids and by GC-IT/MS for fatty acids. In RBL-2H3 cells stimulated either with calcium ionophore or IgE/antigen, the hydromethanolic extract significantly decreased β-hexosaminidase release until the concentration of 2.08 mg/mL, without compromising cellular viability. No effect was found on lipoxygenase. Concerning extract composition, eight organic acids and five fatty acids were determined for the first time. Malonic acid (80%) and α-linolenic acid (27%) were the main compounds in each class. Overall, this study shows promising results, substantiating for the first time the utility of intake of *E. plantagineum* bee pollen to prevent allergy and ameliorate allergy symptoms, although a potentiation of an allergic response can occur, depending on the dose used.

## 1. Introduction

The prevalence and incidence of allergies have increased dramatically in the industrialized world in the past few years [1]. In some individuals the interaction of environmental and genetic factors can lead to the development of allergic disorders following allergen exposure [2]. Allergic diseases are traditionally referred to as immediate or type I hypersensitivity reactions, immunoglobulin (Ig) E being a critical factor. IgE is involved in allergic inflammation, especially in early phase response, but it may also be implicated in the late phase response [3]. The molecular and cellular mechanisms mediating the allergic inflammatory cascade involve multiple mediators, cell types and pathways. Cross-linking by allergen of IgE affixed to high-affinity receptors (FcεRI) on mast cells and basophils triggers degranulation and the release of preformed inflammatory mediators (important to the early phase response), subsequently initiating the synthesis and the release of lipid mediators and cytokines (which may contribute to the late phase response) [4]. Thus, a strategy to prevent IgE-induced mast cell degranulation is central to the discovery of drugs for allergic diseases [5]. Allergens that elicit IgE responses are mostly proteins or glycoproteins with molecular masses of 5–80 KDa, which easily elute from breathable particles and are highly immunogenic [6].

Bee pollen is a traditional product for human consumption and has also been used in folk and complementary medicine to alleviate colds, ulcers, anemia and allergies [2]. Though this product can cause allergic reactions in sensitized individuals, in non-sensitized ones it is a source of antioxidant, anti-inflammatory and anti-allergic compounds, due to its high content of phenolics, such as flavonoids [1]. In recent years, natural antioxidants found in plants have drawn interest due to their presumed nutritional and therapeutic value [7]. *Echium plantagineum* L. bee pollen hydromethanolic extract, previously studied by our group, showed a high content of polyphenols and demonstrated antioxidant and anti-inflammatory activities, being devoid of alkaloids [8,9].

The aim of this study was to determine the effects of *E. plantagineum* bee pollen hydromethanolic extract on rat basophilic leukemic cells (RBL-2H3) degranulation after being subjected to two different stimuli: calcium ionophore A23187 and the IgE/antigen anti-2,4-dinitrophenylated IgE/2,4-dinitro-phenylated-albumin (anti-DNP IgE/DNP-BSA). Additionally, the effect of the extract on lipoxygenase activity, an enzyme involved in the production of leukotrienes during the allergic response [10] was evaluated in a cell- free system. As far as we know, this is the first report of the anti-allergic potential *E. plantagineum* bee pollen. 

This study also aimed to provide further insights on *E. plantagineum* bee pollen metabolic profile, namely its organic and fatty acids. Organic acids are implicated in plant metabolism, including energy production, formation of precursors for amino acids and fatty acids biosynthesis and, at the whole plant level, in modulating adaptation to the environment [11]. The organic and fatty acids composition of this matrix is described for the first time.

## 2. Results and Discussion

### 2.1. Identification and Quantification of Organic Acids by HPLC-UV

**Figure 1:**
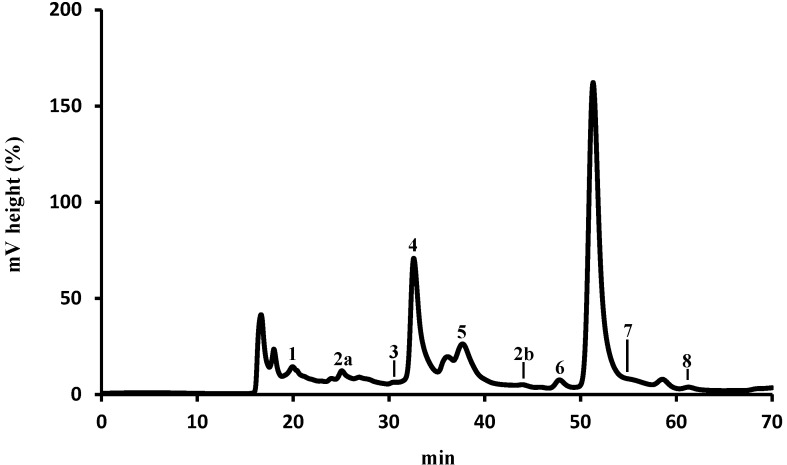
HPLC-UV organic acids profile of *E. plantagineum* hydromethanolic extract. Detection at 214 nm. Identity of compounds as in Table 1.

The organic acids composition varies depending upon the species, age of the plant and the vegetable tissue. In *E. plantagineum* bee pollen hydromethanolic extract eight organic acids were identified: oxalic, aconitic, citric, pyruvic, malonic, shikimic, acetic and fumaric acids (Figure 1).

The total organic acid content of *E. plantagineum* bee pollen was *ca*. 10 g/kg (Table 1), malonic acid (5) being the main compound (*ca.* 80% of total identified organic acids). Fumaric acid (8) was the minor compound (*ca*. 0.005% of the total acids). 

Malonic and acetic acids are the precursors for fatty acids biosynthesis. Furthermore, shikimic and malonic acids are precursors of phenolic compounds, a heterogeneous group from a metabolic point of view, presenting antioxidant, anti-inflammatory and anti-allergic activities [12]. 

**Table 1:** Organic acids composition of *E. plantagineum* bee pollen.

	Organic Acid	mg/kg (Dry Pollen) ^a^	%
**1**	Oxalic	51.05 ± 0.98	0.50
**2a + 2b**	Aconitic	6.89 ± 0.26	0.07
**3**	Citric	10.24 ±0.05	0.10
**4**	Pyruvic	890.27 ± 0.85	8.69
**5**	Malonic	8152.87 ± 0.37	79.57
**6**	Shikimic	2.20 ± 0.43	0.02
**7**	Acetic	1132.02 ± 111.33	11.05
**8**	Fumaric	0.48 ± 0.02	0.005
	**Total**	**10245.54 ± 114.29**	

^a^ Results are expressed as mean of three determinations ± standard deviation.

### 2.2. Identification and Quantification of Fatty Acids by GC-IT/MS

**Figure 2:**
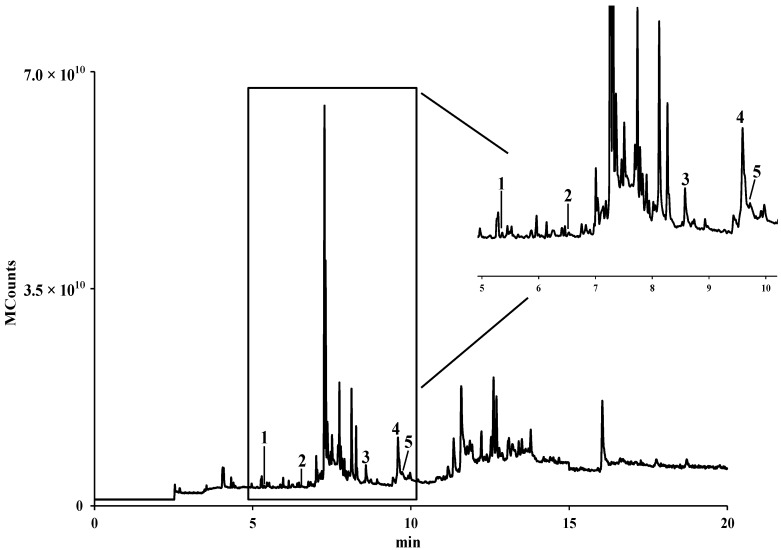
GC-IT/MS fatty acids profile of *E. plantagineum* hydromethanolic extract. Identity of compounds as in Table 2. All compounds correspond to their trimethylsilyl (TMS) derivatives.

It is known that pollen contains all nutrients, including lipids that are necessary for plant growth and development [13]. Five fatty acids were determined in *E. plantagineum* bee pollen hydromethanolic extract, after purification, performed in order to protect the equipment and facilitate identification (Figure 2).

The fatty acids content was *ca*. 690 mg/kg, palmitic acid being the major compound (*ca.* 57%) followed by α-linolenic acid, which represented *ca*. 27% of the identified compounds (Table 2).

**Table 2:** Fatty acids composition of *E. plantagineum* bee pollen.

	Fatty Acid	mg/kg (Dry Pollen) ^a^	%
**1**	Capric (C10:0)	53.0 ± 1.7	7.67
**2**	Lauric (C12:0)	27.1 ± 2.6	3.93
**3**	Palmitic (C16:0)	391.6 ± 19.3	56.72
**4**	α-Linolenic (C18:3)	183.9 ± 18.6	26.64
**5**	Stearic (C18:0)	34.8 ± 2.8	5.04
	**Total**	**690.4 ± 45.0**	

^a^ Results are expressed as mean of three determinations ± standard deviation.

Several fatty acids were previously described in the oils obtained from *E. plantagineum* seeds [14] and leaves [15]. Although the fatty acids profile of bee pollen hydromethanolic extract is different from the ones previously described in *E. plantagineum* seeds and leaves oils, α-linolenic acid is a representative fatty acid in all plant materials. This unsaturated lipid is important because it is metabolized to longer polyunsaturated fatty acids that are known to have beneficial effects on inflammatory and autoimmune diseases [16].

### 2.3. Effect on RBL-2H3 Cells Viability and Degranulation

**Figure 3:**
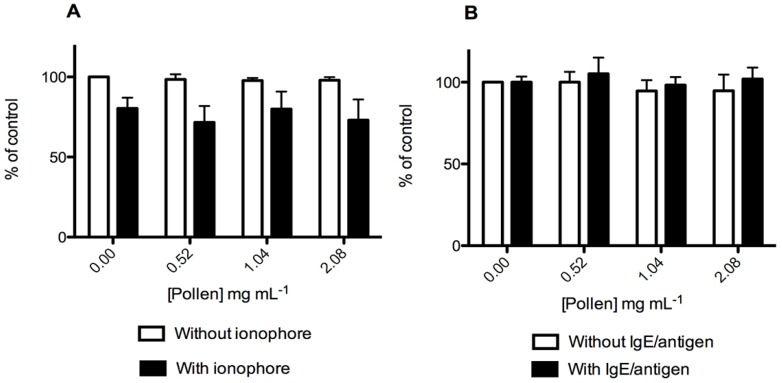
Effect of *E. plantagineum* bee pollen extract in RBL-2H3 basophils viability, assessed by MTT reduction assay. Values show mean ± SEM of three independent assays performed in duplicate with calcium ionophore (or extract only) and six independent assays performed in duplicate with IgE-DNP – DNP-BSA (or extract only). (**A**) Cells were pre-exposed to the extract for 15 min, followed by 30 min co-exposure to 500 ng/mL calcium ionophore A23187; (**B**) Cells were pre-exposed to 100 ng/mL anti-DNP IgE for 16 h, followed by 1 h extract co-exposure to 100 ng/mL DNP-BSA.

The effects of the extract on cellular viability were evaluated by the MTT reduction assay. As it can be seen in Figure 3, the extract was not cytotoxic to RBL-2H3 cells in the range of concentrations used to evaluate its anti-allergic potential (0.52 to 2.08 mg/mL). The extract by itself did not affect cell viability till the concentration of 8.33 mg/mL (data not shown). However, basophils pre-exposed to the hydromethanolic extract for 15 min followed by co-exposition for 30 min with calcium ionophore (500 ng/mL) suffered a significant viability decrease for concentrations above 4.17 mg/mL (data not shown). So, as ionophore A23187 is highly selective for calcium [17] it can be assumed that the interaction of the compounds present in the extract with the calcium ionophore leads to cellular calcium overload, compromising cellular viability for the higher extract concentrations tested.

MTT reduction assay was also performed for RBL-2H3 cells treated with IgE/antigen in the presence and in the absence of the extract. The IgE/antigen treatment by itself did not affect basophils viability, either in the presence or absence of the extract (Figure 3B).

In this study we used a crude extract to evaluate the anti-allergic potential of *E. plantagineum* bee pollen since bee pollen is consumed in folk and complementary medicines as a whole [2]. So the extraction method used allowed the recovery of a wide variety of compounds, in order to guarantee that the compounds that have an activity *in vivo* were not lost during the extraction process. The purification of the extract was not attempted as the effect of bee pollen cannot be completely described from the activities of a specific group of compounds since synergic and/or antagonic effects between all the constituents can occur. 

Because the extract was not purified we needed to use relatively high concentrations to obtain a response in the *in vitro* assay. However, it is usually seen that it is not possible to directly extrapolate concentrations used in *in vitro* assays to the *in vivo* situation [18]. 

The extract revealed to be composed by several organic and fatty acids. These compounds could affect the results of degranulation since degranulation phenomenon is influenced by pH [19]. However, as the pH range of the medium used in the assays was buffered to pH 6.8–7.2, the amounts of acids in the extracts were not sufficient to exceed the buffering capacity of the culture medium and so degranulation was not affected. 

**Figure 4:**
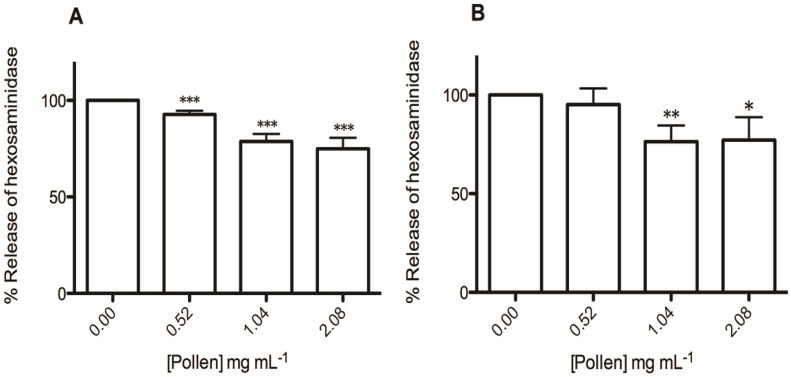
Effect of *E. plantagineum* bee pollen extract in β-hexosaminidase release. Values show mean ± SEM of three independent assays performed in duplicate with calcium ionophore stimulus and six independent assays performed in duplicate using IgE-DNP – DNP-BSA stimulus. (**A**) % of β-hexosaminidase release after RBL-2H3 exposure to ionophore; *** *p* < 0.001. (**B**) % of β-hexosaminidase release after RBL-2H3 pre-exposure to anti-DNP IgE, followed by DNP-BSA exposure; * *p* < 0.05; ** *p* < 0.01.

The rat basophilic leukemic (RBL-2H3) cell line has been widely used for allergy and immunological research [5,20,21]. The release of β-hexosaminidase enzyme, a marker of degranulation in mast cells and basophils, allows estimating the anti-allergic potential of new drugs *in vitro* [20]. In a first approach we used calcium ionophore A23187 to elicit degranulation of RBL-2H3 cells. Artificial activation of basophils by calcium ionophore A23187 is routinely used in the research of exocytosis to bypass the molecular events that are associated with the excitation of FcεRI receptors [22]. With this stimulus the extract inhibited β-hexosaminidase release in a dose-dependent manner (*p* < 0.001) (Figure 4A). Additionally, β-hexosaminidase release was further investigated using higher extract concentrations. It was observed that the extract decreased β-hexosaminidase release at 4.17 mg/mL (64.42% ± 2.55%, *p* < 0.001), but at this concentration the cellular viability was significantly affected (data not shown). The subsequent increase of β-hexosaminidase release at 8.33 mg/mL (90.98% ± 6.58%) may result not only from cell degranulation, but also from enzyme leakage by unviable cells with disrupted membrane integrity [23]. 

The anti-allergic potential of the extract was also evaluated using IgE/antigen stimulus, since basophils possess high-affinity IgE receptors [24]. As referred above, the cross-linking of FcεRI-bound IgE by allergens plays a pivotal role in IgE-mediated allergy [4]. Using this allergic stimulus the co-exposition with the extract significantly inhibited the DNP-BSA-induced β-hexosaminidase secretion in IgE-sensitized RBL-2H3 cells at 1.04 and 2.08 mg/mL (23.61% ± 8.15%, *p* < 0.01, and 22.84% ± 11.57%, *p* < 0.05, respectively) (Figure 4B). As observed with the calcium ionophore, when higher concentrations of the extract were assayed an increase in β-hexosaminidase release was observed (data not shown). However, with this stimulus there was no observable decrease of cell viability. So, we can argue that at higher concentrations the extract demonstrated a potential allergic effect in IgE sensitized basophils. In a previous study the extract has demonstrated a pro-oxidant potential at these same concentrations [9]. Thus, it may be speculated that the generation of reactive oxygen species influenced the signaling pathways evoked by the IgE/antigen stimulus, leading to enhanced degranulation. This behavior has already been observed for compounds with pro-oxidant activity [25]. 

The anti-allergic potential demonstrated by the extract can be attributed to several compounds, namely the polyphenols previously described [9] and the fatty acids reported herein. Palmitic and α-linolenic acids, the two major fatty acids identified in the extract, already have reported anti-allergic effects. In fact, among the fatty acids found in the extract, α-linolenic acid has been demonstrated to reduce the release of inflammatory mediators, including histamine and prostaglandin E2 [26]. Moreover, epidemiological studies show that high intake of α-linolenic and palmitic acids during pregnancy may decrease the risk of asthma of the offspring [27]. So, the anti-degranulation effects observed in the model used in the present study can result partially from the presence of fatty acids in the extract.

Concerning the polyphenols, these compounds are well known for their anti-allergic potential [28]. In fact, the tested extract contains derivatives of quercetin and kaempferol, which have inhibitory effect on degranulation of mast-cells and basophils [1]. This effect is thought to result from two mechanisms: one by which polyphenols impact allergen-IgE complex formation and another through which they impact on the binding of this complex to their basophil FcεRI [1]. This can be explained by the formation of insoluble complexes between the polyphenols and potential allergenic proteins, rendering them to be hypoallergenic [1]. Nevertheless, the extract was more effective in inhibiting degranulation when the calcium ionophore was used (the effective concentrations ranged between 0.52 mg/mL and 2.08 mg/mL, while in the assay with IgE/antigen the effective concentrations varied between 1.04 mg/mL and 2.08 mg/mL), suggesting that the compounds present in the extract also affected the increase of cytoplasmic calcium, an event that immediately precedes degranulation. 

In this work a direct effect of organic acids in degranulation could not be established. However, as malonic and acetic acids are precursors of fatty acids and also of flavonoids (together with shikimic acid), the determination of organic acids was considered important for the characterization of the metabolic profile of this matrix.

In order to clarify whether the effects of the extract were due to the inhibition of hexosaminidase release, and not a false positive resulting from the inhibition of β-hexosaminidase activity, we tested the effect of the extract on β-hexosaminidase activity using a basophil cell lysate as source of β-hexosaminidase. As the extract did not inhibited β-hexosaminidase enzymatic activity (data not shown), the β-hexosaminidase results observed in basophils insulted with an allergic stimulus are due to the extract effects on degranulation.

Considering the results obtained with the extract, although the percentage of inhibition of degranulation was not very high, it was significantly different from the control, justifying the traditional use of bee pollen in allergic diseases.

### 2.4. Effect on Soybean Lipoxygenase Activity

Lipoxygenases are enzymes involved in the arachidonic acid pathway, which catalyze the peroxidation of polyunsaturated fatty acids in a selective way. 5-Lipoxygenase metabolizes free arachidonic acid to 5-hydroperoxy-eicosatetraenoic acid (5-HPETE), which is further metabolized to leukotrienes. These compounds are pivotal lipid mediators in allergy and inflammation [29]. Because of structural and functional similarities with mammalian enzymes, lipoxygenase obtained from soybean is widely accepted as a model for lipoxygenase inhibition studies [30]. In this assay, *E. plantagineum* bee pollen hydromethanolic extract was not able to inhibit the activity of this enzyme. In a previous study, using a cellular model of lipopolysaccharide (LPS)-stimulated macrophages, the extract decreased the levels of arachidonic acid metabolites derived from cyclooxygenase 2, demonstrating that it has anti-inflammatory activity [9]. As during an allergic reaction an inflammatory activity is involved, the extract can contribute to ameliorate the symptoms of allergy, through a mechanism not involving the inhibition of lipoxygenase.

## 3. Experimental Section

### 3.1. Standards and Reagents

Dulbecco’s Phosphate Buffered Saline (DPBS), Dulbecco’s Modified Eagle Medium (DMEM)+GlutaMAX™-I, Earle’s Balanced Salt Solution (EBSS), heat inactivated fetal bovine serum and penicillin+streptomycin (Pen Strep) were obtained from Gibco, Invitrogen™ (Grand Island, NY, USA). Lipoxygenase from *Glycine max* (L.) Merr. (type V-S; EC 1.13.11.12), methyl linolelaidate, N-methyl-N-(trimethylsilyl)trifluoroacetamide (MSTFA), capric, lauric, palmitic, linolenic, stearic, oxalic, aconitic, citric, pyruvic, malonic, shikimic and fumaric acids, 3-(4,5-dimethylthiazol-2-yl)-2,5-diphenyltetrazolium bromide (MTT), methanol, Triton, tris-HCl, dimethyl sulfoxide (DMSO), DL-dithiothreitol (DTT), phenylmethanesulfonyl fluoride (PMSF), calcium ionophore A23187, mouse anti-2,4-dinitrophenylated IgE (anti-DNP IgE), albumin from bovine serum (BSA), 2,4-dinitrophenylated BSA (DNP-BSA) and 4-nitrophenyl N-acetyl-β-d-glucosaminide were purchased from Sigma-Aldrich (St. Louis, MO, USA). The *n*-alkane series (C8–C40) standard was from Supelco (Bellefonte, PA, USA). Sodium chloride was from Merck (Darmstadt, Germany) and acetic and sulfuric acids were from Fisher Scientific (Loughborough, UK). The water was treated in a Milli-Q water purification system (Millipore, Bedford, MA, USA).

### 3.2. Sampling

*E. plantagineum* bee pollen sample was provided by beekeepers in the Spanish Extremadura region, in 2011. The botanical origin was assessed by Paula B. Andrade (Laboratório de Farmacognosia, Faculdade de Farmácia da Universidade do Porto; voucher specimen EP-BP-032011). Bee pollen was stored protected from light, under desiccating conditions to prevent alteration.

### 3.3. Extract Preparation

The extract was prepared as previously described [8]. Briefly, bee pollen (0.2 g) was thoroughly mixed with methanol-water (7:3, 1 mL), ultrasonicated for 1 h and centrifuged at 2,900 *×g* during 10 min (Rotofix 32A, Hettich Lab, Tuttlingen, Germany). The supernatant was evaporated under reduced pressure at 40 °C and the dry residue was stored at −20 °C, protected from light, until use.

### 3.4. Preparation of Standard Solutions

Stock solutions of organic acids standards were prepared in sulfuric acid 0.01 N. Fatty acids and the internal standard (IS) methyl linolelaidate were prepared individually in ethanol and kept at −20 °C until analysis. Calibration solutions were prepared by diluting each stock solution with appropriate amounts of solvent.

### 3.5. HPLC-UV Analysis of Organic Acids

For organic acids analysis the dry residue of the hydromethanolic extract was dissolved in sulfuric acid 0.01 N. The HPLC-UV analysis was carried out as previously reported [31], in a system consisting of an analytical HPLC unit (Gilson, Villiers-le-bel, France) with an ion exclusion column, Nucleogel Ion 300 OA (300 × 7.7 mm; Düren, Germany), in conjunction with a column heating device set at 30 °C. Elution was carried out isocratically, at a solvent flow rate of 0.2 mL/min, with sulfuric acid 0.01 N. The sample volume was 20 μL. Detection was performed with a UV-Vis detector set at 214 nm. Organic acids quantification was achieved by the absorbance recorded in the chromatograms relative to external standards. The data were processed on a Clarity Software system (Data Apex, Prague, Czech Republic). Analyses were performed in triplicate.

### 3.6. Fatty Acids Purification and Derivatization

The extract was purified before fatty acids derivatization. The dry residue of the hydromethanolic extract was thoroughly mixed with acidic water (pH 2 with HCl) and applied to an Amberlite XAD-2 (Fluka Chemicals, Steinheim, Germany: pore size 9 nm, particle size 0.3–1.2 mm) column. Sugars and other polar compounds were eluted with the aqueous solvent. The retained compounds were eluted with ethanol and the IS methyl linolelaidate (100.00 µg/mL final concentration) was added. The volume was completed to 2.00 mL with ethanol. Fatty acid derivatization was carried out as previously described [32]. Briefly, an aliquot of 50 μL of purified extract solution was transferred to a glass vial, the solvent was evaporated under nitrogen stream and 50 μL of derivatization reagent (MSTFA) were added to the dried residue. The vial was capped, vortexed and heated for 20 min in a dry block heater maintained at 60 °C. Analyses were performed in triplicate.

### 3.7. GC-IT/MS Analysis of Fatty Acids

Samples were analyzed using a Varian CP-3800 gas chromatographer coupled to a Varian Saturn 4000 mass selective ion trap detector (Palo Alto, CA, USA) and a Saturn GC-MS workstation software version 6.8. A VF-5 ms (30 m × 0.25 mm × 0.25 µm) column (Varian) and a CombiPAL automatic auto sampler (Varian) were used. The injector port was heated to 250 °C. Injections were performed in split mode, with a ratio of 1/40. The carrier gas was helium C-60 (Gasin, Leça da Palmeira, Portugal), at a constant flow of 1 mL/min. The ion trap detector was set as follows: transfer line, manifold and trap temperatures were 280, 50 and 180 °C, respectively. The mass ranged from 50 to 600 *m/z*, with a scan rate of 6 scan/s. The emission current was 50 µA and the electron multiplier was set in relative mode to auto tune procedure. The maximum ionization time was 25.000 µs, with an ionization storage level of 35 *m/z*. The injection volume was 2 µL and the analysis was performed in Full Scan mode. The oven temperature was set at 100 °C for 1 min, then increasing 20 °C/min to 250 °C and held for 2 min, 10 °C/min to 300 °C and held for 10 min. All mass spectra were acquired in electron impact (EI) mode. Ionization was maintained off during the first 4 min to avoid solvent overloading. Compounds were identified by comparison of retention times and MS fragmentation pattern with those of standards analyzed under the same conditions and a mass spectra database search was performed using the National Institute of Standards and Technology (NIST) MS 05 spectral database. In addition, the retention index (RI) was experimentally calculated and the values were compared with those reported in the literature for GC columns with 5%-phenyl-95%-dimethylpolysiloxane [33,34]. For the RI determination, an *n*-alkanes series (C8–C40) was used.

### 3.8. Cell Culture and Treatments

The rat basophil cell line RBL-2H3 was purchased from American Type Culture Collection (ATCC^®^; LGC Standards S.L.U., Barcelona, Spain). Cells were grown in DMEM+GlutaMAX ™ – I supplemented with 10% heat inactivated fetal bovine serum, 100 U/mL penicillin and 100 μg/mL streptomycin, under 5% CO_2_ at 37 °C, in humidified air. For the anti-allergic assays cells were plated at 3 × 10^5^ cells/ mL in 24-wells plates (1 mL/well) and treated after reaching confluence. 

In the assay using calcium ionophore as stimulus, cells were pre-treated with the extract (dissolved in culture medium) 15 min prior to the addition of the ionophore (1 µM in EBSS + 0.1% BSA) and maintained in culture for another 30 min. Then the supernatant was collected and used for the determination of β-hexosaminidase release, whereas the MTT cell viability assay was performed in adherent cells. Three independent assays were performed in duplicate. In another experiment cells were sensitized with anti-DNP IgE (100 ng/mL) during 16 h, then stimulated for 1 h with DNP-BSA (100 ng/mL in EBSS + 0.1% BSA). Cells were co-incubated with the extract throughout the assay. β-Hexosaminidase release and MTT assays were performed as in the assay with calcium ionophore. Six independent assays were performed in duplicate. The effect of the extract on β-hexosaminidase release in the absence of stimuli (calcium ionophore or IgE/antigen) was simultaneously tested in every assay performed with and allergic stimulus. 

### 3.9. MTT Reduction Assay

Cellular viability was assessed by the reduction of MTT to formazan, according to a previously described method [9], with some modifications. After cell treatment, culture medium removal and cells washing with DPBS, cells in 24-wells plates were incubated with 1 mL/well of MTT (0.5 mg/mL), for 30 min at 37 °C. Supernatant was then discarded, formazan was solubilized in DMSO (1 mL) and quantified by measurement of optical density at 550 nm, using a microplate reader (Multiskan ASCENT Thermo^®^, Vantaa, Finland). Results are expressed as percentage of control (without allergic stimulus). Three independent assays were performed in duplicate with calcium ionophore (or extract only) and six independent assays were performed in duplicate with IgE-DNP – DNP-BSA (or extract only). 

### 3.10. Quantification of Released β-Hexosaminidase

To assess the effect of the extract in RBL-2H3 cells degranulation, β-hexosaminidase was quantified according to a previously published method [35], with some modifications. Briefly, 30 μL of supernatant was incubated with 50 μL of 1.3 mg/mL 4-nitrophenyl N-acetyl-β-d-glucosaminide (in 0.2 M citric acid pH 4.5) for 1 h at 37 °C. This β-hexosaminidase reaction was stopped by the addition of 80 μL of NaOH 0.5 M and absorbance was measured at 405 nm using a microplate reader (Multiskan ASCENT Thermo^®^). Three independent assays were performed in duplicate in the assay with calcium ionophore (or extract only) and six independent assays were performed in duplicate in the assay with IgE-DNP – DNP-BSA (or extract only).

### 3.11. β-Hexosaminidase Inhibitory Activity

Beyond avoiding β-hexosaminidase release, the extract may directly inhibit β-hexosaminidase enzymatic activity. The inhibition of β-hexosaminidase activity was checked by the following assay [35]: after culture, cells were lysed by incubation with lysis buffer (50 mM Tris-HCl pH 8.0, 150 mM NaCl, 1% Triton X-100) for 1 h, at 0 °C. Lysates were centrifuged at 8,000 *×g* for 5 min. The resulting supernatants were used to determine β-hexosaminidase activity in the presence and in the absence of the extract, as described in the previous section. Three independent assays were performed in duplicate.

### 3.12. Lipoxygenase Inhibition Assay

The oxidation of linoleic acid by lipoxygenase to the conjugated diene 13-hydroperoxy linoleic acid was followed by measuring the absorbance at 234 nm on an UV/vis spectrophotometer (Helios Alpha, Cambridge, UK). The assay was performed by adding 20 μL of extract dissolved in phosphate buffer (pH 9.0), 1 mL of phosphate buffer and 20 μL of a soybean lipoxygenase solution (500 U). After 5 min incubation, at room temperature, the reaction was started by the addition of 50 µL sodium linoleate (2 mM in ethanol). The reaction time was 3 min and the inhibition of the enzyme activity was calculated by comparing the reaction rate with the control (without extract) [35].

### 3.13. Statistical Analysis

Data are expressed as the mean ± standard error of the mean (SEM) of at least three independent experiments. Two-way ANOVA and Bonferroni’s test, as post-hoc test, were used to determine the interaction of the stimulus and the extract in MTT reduction assay. Two-tailed t paired test was used to determine the statistical significance in comparison to control in β-hexosaminidase release assay. *p* Values of 0.05 or less were considered statistically significant. Statistical analyses were performed using GraphPad Prism 5 Software (San Diego, CA, USA).

## 4. Conclusions

Presently there is a high interest in the ingestion of bee pollen as a health-promoting natural product, due to its flavonoids and unsaturated fatty acids contents. Notwithstanding the fact that susceptible individuals should be aware of the risk of allergic reactivity (and cross-reactivity) occurrence by pollen exposure, this study presents an interesting perspective for *E. plantagineum* bee pollen intake to prevent allergy and ameliorate allergy symptoms. The results demonstrated that the hydromethanolic extract was effective in inhibiting basophils degranulation under an allergic stimulus, as ascertained by the levels of β-hexosaminidase released. This anti-allergic activity may be associated with its chemical composition, namely its polyphenolic compounds and unsaturated fatty acids. However, as the extract at the highest tested concentrations showed some deleterious effects on insulted cells, further studies concerning bee pollen extract activity and bioavailability are necessary before using it as a phytomedicine in allergic diseases. 
